# A Neonate With Diabetes Mellitus, Congenital Hypothyroidism, and Congenital Glaucoma

**DOI:** 10.7759/cureus.29488

**Published:** 2022-09-23

**Authors:** Praveen Kumar Boddu, Pradeep Kumar Velumula, Saima Sharif, Bajaj Monika

**Affiliations:** 1 Neonatology, Central Michigan University College of Medicine, Mount Pleasent, USA; 2 Neonatology, MercyOne Waterloo Medical Center, Waterloo, USA

**Keywords:** glis3 gene mutation, congenital hypothyroidism, familial neonatal diabetes mellitus, hyperglycemia in children, preterm neonate, primary congenital glaucoma

## Abstract

Neonatal diabetes mellitus (NDM) is a rare condition with more than 20 monogenic genes associated with it. *GLIS3* gene-encoded GLI similar protein 3, as a transcription factor, is involved in the development of the pancreas, liver, kidneys, eye, and thyroid. We report a preterm female neonate with coarse facial features and hyperglycemia, later diagnosed with neonatal diabetes mellitus, congenital hypothyroidism (CH), congenital glaucoma (CG), and renal cysts, secondary to *GLIS3* gene mutation. It is a rare genetic disorder involving multiple organ systems with progressive development of symptoms requiring long-term surveillance and management.

## Introduction

Neonatal diabetes mellitus (NDM) is a rare genetic disorder with an estimated incidence of one in 20,000 to one in 500,000 [1]. NDM is reported more commonly in the Middle Eastern region [2]. Syndromic NDM constitutes 10% of this rare patient group [3]. NDM with congenital hypothyroidism (CH) is a rare condition caused by homozygous or compound heterozygous mutations in *GLIS3* gene. *GLIS3* belongs to the GLIS subfamily of Krüppel-like zinc finger proteins and functions as an activator and repressor of transcription [4]. The protein is involved in the development of the pancreas, thyroid, eyes, liver, and kidneys. In this case report, we present a neonate diagnosed with neonatal diabetes mellitus due to a compound heterozygous mutation in *GLIS3* gene.

## Case presentation

A preterm diamniotic-dichorionic twin neonate delivered at 32 weeks gestational age was admitted to neonatal intensive care unit (NICU) for management of prematurity and respiratory distress after birth. The infant's mother is a 29-year-old, gravida 4, para 4, who had regular prenatal care during pregnancy. The mother denies history of abortions. The parents of the infant are first cousins and are of Middle Eastern origin (Yemeni). Her prenatal laboratory work was negative for group B streptococcus, HIV, hepatitis B surface antigen, syphilis, gonorrhea, and chlamydia. Fetal anomaly scan showed no abnormalities. The infant was delivered by cesarean section for category II fetal heart tracing at 32 weeks gestational age. Appearance, pulse, grimace, activity, and respiration (APGAR) scores were 6 and 8 at one and five minutes, respectively. She was started on continuous positive airway pressure (CPAP) for respiratory distress and transferred to neonatal intensive care unit for further management. The neonate was small for gestational age with birth weight of 1275 g (fourth percentile), head circumference of 27 cm (fourth percentile), and length of 38 cm (fourth percentile). Vital signs were stable. Physical examination was significant for tachypnea with retractions, coarse facial features, protruding tongue, and a wide-open anterior fontanelle.

NICU course

The infant was started on CPAP, and the respiratory support was slowly weaned to room air by day 7 after birth. A chest X-ray done at the time of admission was suggestive of respiratory distress syndrome. Following sepsis screen, she was started on antibiotics, which were discontinued as the blood cultures showed no growth. A two-dimension (2D) echocardiogram showed moderate-size patent ductus arteriosus and patent foramen ovale with left to right shunt. The infant's blood glucose levels were found to be above the reference range from first day after birth. The highest blood glucose value noted during the admission was 480 mg/dl. On sixth day after birth, insulin infusion was started at a dose of 0.01-0.02 units/kg/hour, which was later weaned to subcutaneous insulin (Lantus) at a dose of 0.05 units. Serum insulin level, done while the infant was receiving insulin therapy, was 8.39 µIU/mL (reference range: 1.9-23.0 µIU/mL), and C-peptide level was 0.2 ng/mL (1.1-4.4 ng/mL) suggestive of exogenous insulin administration and deficiency of endogenous insulin. The newborn genetic screen sent at 24 hours after birth resulted positive for congenital hypothyroidism, which was confirmed with serum thyroid profile (elevated thyroid-stimulating hormone {TSH} and low free T4). She was started on levothyroxine 25 µg/day. There were no similar concerns in the other twin who received routine preterm care and was discharged home.

On further enquiry, the mother gave a family history of one of the siblings being diagnosed with neonatal diabetes mellitus and congenital hypothyroidism associated with *GLIS3* gene mutation. Genetics team was consulted. A chromosomal microarray was sent, which identified a homozygous deletion at 9p24.2 that was 38.8 Kb in size, including exons 10 and 11 of the *GLIS3* gene. Since the *GLIS3* gene mutation is associated with other organ involvement, the infant was further investigated. A head ultrasound done in the first week of life showed grade I intraventricular hemorrhage, which resolved on the follow-up ultrasound scan. Her liver function tests were within the reference range for age, and liver ultrasound noted a focal echogenic focus suggestive of focal calcification. Her renal function tests were within reference range, and renal ultrasound noted multiple round anechoic lesions in the bilateral kidneys, the largest on the right kidney measuring 5 × 5 × 6 mm in the right lower pole and the largest on the left kidney measuring 3 × 3 × 4 mm in the left lower pole. Ophthalmological assessment showed bilateral corneal clouding with raised intraocular pressure. Treatment with timolol and latanoprost eye drops was started for congenital glaucoma (CG). Later, she underwent trabeculotomy in both eyes. She was discharged on 104 days of age with follow-up appointments with genetics, endocrinology, gastroenterology, and nephrology services.

During the follow-up visit at five months post menstrual age, the infant was at 10th percentile for weight. She continues to receive subcutaneous insulin for diabetes mellitus, levothyroxine for congenital hypothyroidism, and timolol, latanoprost, and dorzolamide eye drops for glaucoma. Her most recent glycosylated hemoglobin (HbA1c) was 5.6%. She underwent serial renal ultrasounds for renal cysts, and on the latest renal ultrasound, the cyst on the right side was found to be slightly increased in size. Her renal function tests were still within reference range. She was developmentally appropriate for age.

## Discussion

Neonatal diabetes mellitus (NDM) is a rare condition with a relatively higher incidence in the Middle Eastern population [2,5]. NDM could be transient, permanent, or a part of syndromic presentation. There are more than 20 monogenic genes that are associated with NDM [3,6]. NDM secondary to genetic mutations could be from either abnormal development of the pancreas, abnormal β-cell function, or β-cell destruction [7]. The mutation of *GLIS3* gene is one of the monogenic gene mutations associated with NDM. GLI similar protein 3 is a zinc finger protein and can act as an activator or repressor of transcription. It plays a crucial role in the development of the pancreas, thyroid, eyes, liver, kidneys, and other organ systems [4,8-10]. Senée and colleagues in 2006 described, for the first time, a new syndrome in an infant diagnosed with NDM and CH associated with *GLIS3* gene mutation [11]. So far, 22 cases have been reported in the literature, and they all exhibit phenotypic variation [11,12].

*GLIS3* gene mutation predominantly presents with NDM and CH. Other systems commonly involved with this mutation are the eyes (CG), kidneys (renal cysts), and liver (hepatomegaly, hepatitis, hepatic fibrosis, cirrhosis, and cholestasis) [12]. Some less frequently associated features include dysmorphism, exocrine pancreatic insufficiency, osteopenia, sensorineural deafness, skeletal anomalies, choanal atresia, patent ductus arteriosus, atrial septal defect, and psychomotor delays [2]. Similar to our case, deletions in exons 10 and 11 have been reported in a female child of Yemeni origin [13]. This patient presented with congenital hypothyroidism, congenital glaucoma, renal cysts, developed hepatic fibrosis, and exocrine pancreatic insufficiency [12,13].

**Figure 1:**
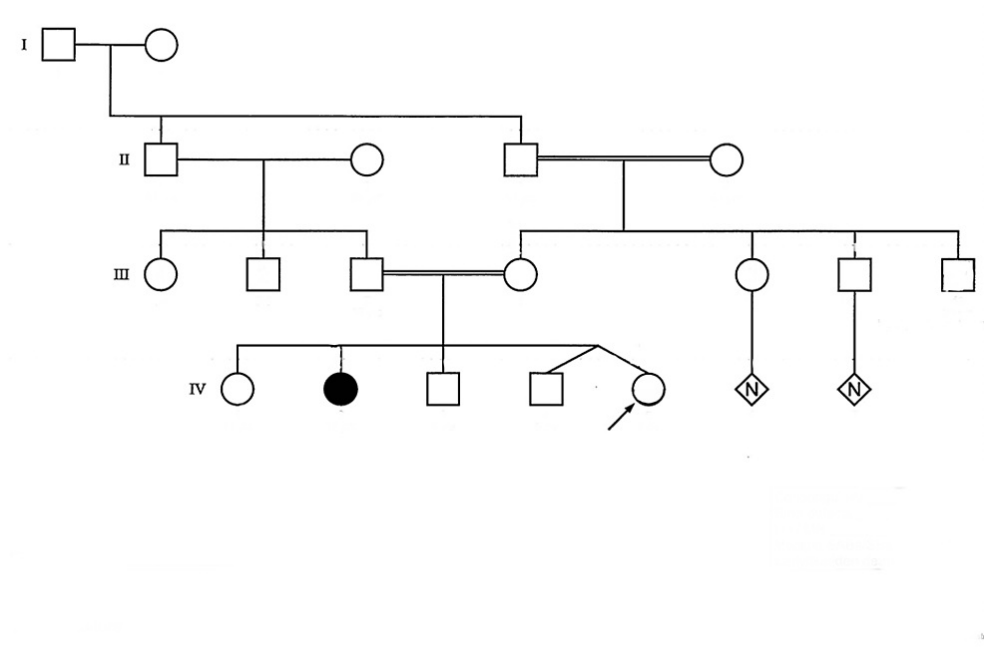
Pedigree chart The arrow points to the patient in the discussion. The solid black circle indicates the patient's sister who was diagnosed with similar genetic mutation

The infant in our case report presented with hyperglycemia in the early neonatal period and on further investigation was diagnosed with NDM, CH, and CG associated with *GLIS3* gene mutation. There were multiple renal cysts in bilateral kidneys albeit with normal renal function. Her 11-year-old sibling (Figure 1) has a similar gene mutation and was diagnosed with NDM, CH, and CG. He is developmentally delayed and awaiting renal and liver transplantation for end-stage renal and liver failure.

## Conclusions

Our case underscores the importance of genetic work-up and counseling in infants diagnosed with neonatal diabetes. In these infants, *GLIS3* gene mutation should be considered in the differential diagnosis. This gene mutation may involve multiple organ systems, and a multidisciplinary management of the patients diagnosed with this gene mutation is warranted.
